# Tumor-associated macrophages in anti-PD-1/PD-L1 immunotherapy for hepatocellular carcinoma: recent research progress

**DOI:** 10.3389/fphar.2024.1382256

**Published:** 2024-06-18

**Authors:** Ziwei Li, Dongyu Duan, Li Li, Dan Peng, Yue Ming, Rui Ni, Yao Liu

**Affiliations:** Department of Pharmacy, Daping Hospital, Army Medical University, Chongqing, China

**Keywords:** tumor-associated macrophages (TAMs), hepatocellular carcinoma (HCC), PD-1/PD-L1, T cell, immunotherapy

## Abstract

Hepatocellular carcinoma (HCC) is one of the cancers that seriously threaten human health. Immunotherapy serves as the mainstay of treatment for HCC patients by targeting the programmed cell death protein 1/programmed cell death 1 ligand 1 (PD-1/PD-L1) axis. However, the effectiveness of anti-PD-1/PD-L1 treatment is limited when HCC becomes drug-resistant. Tumor-associated macrophages (TAMs) are an important factor in the negative regulation of PD-1 antibody targeted therapy in the tumor microenvironment (TME). Therefore, as an emerging direction in cancer immunotherapy research for the treatment of HCC, it is crucial to elucidate the correlations and mechanisms between TAMs and PD-1/PD-L1-mediated immune tolerance. This paper summarizes the effects of TAMs on the pathogenesis and progression of HCC and their impact on HCC anti-PD-1/PD-L1 immunotherapy, and further explores current potential therapeutic strategies that target TAMs in HCC, including eliminating TAMs in the TME, inhibiting TAMs recruitment to tumors and functionally repolarizing M2-TAMs (tumor-supportive) to M1-TAMs (antitumor type).

## 1 Introduction

Hepatocellular carcinoma (HCC) is a primary liver malignancy clinically characterized by a relatively high mortality, insidious onset, rapid progression, early recurrence, and poor prognosis. Since it is technically challenging to diagnose HCC at a very early stage, most patients present with late-stage HCC at initial diagnosis and thus miss the ideal time for hepatectomy or radiofrequency ablation (Bray et al., 2018). In addition, HCC is not sensitive to chemotherapy and has fewer targeted benefits, which makes immunotherapy a source of hope for long-term survival. Current immunotherapies mainly focus on HCC treatments with proved anticancer efficacy, primarily including immune checkpoint inhibition, vaccination, and adoptive T-cell therapy. Particularly, the immunotherapy targeting programmed cell death protein 1/programmed cell death 1 ligand 1 (PD-1/PD-L1) is the latest treatment available for HCC patients (Balar and Weber, 2017). Anti-PD-1/PD-L1 immunotherapy is a life-changing regimen for HCC patients as it substantially improves the overall survival (OS) and progression-free survival (PFS) (Hamanishi et al., 2016). However, clinical data demonstrate that the treatment effect of solid tumors treated with anti-PD-1/PD-L1 monoclonal antibodies is far from satisfactory, with only a few patients benefiting from anti-PD-1 therapy, and most of them experience drug resistance and disease progression in the process of anti-PD-1/PD-L1 immunotherapy. The efficacy of anti-PD-1/PD-L1 immunotherapy is associated with many factors, including (i) insufficient T cell infiltration into tumor tissue, (ii) the immunosuppressive tumor microenvironment (TME) consisting of bone marrow-derived immunosuppressive cells and regulatory T cells (Treg), (iii) physiological barriers such as vascular malformations, high interstitial fluid hydrostatic pressure, and dense extracellular matrix (ECM) that impede effective accumulation and penetration of anti-PD-1 (aPD-1) antibodies into tumor tissue, and (iv) immunosuppressive activities of tumor-associated macrophages (TAMs) (Maute et al., 2015; Pitt Jonathan et al., 2016; Sharma et al., 2017; Binnewies et al., 2018; Yang and Hu, 2019).

Particularly, TAMs represent about 40% of tumor-infiltrating inflammatory cells, serving as key players in the development and progression of HCC, and are associated with a poorer prognosis for HCC. TAMs are infiltrating macrophages in tumor tissue, which are mainly differentiated from monocytes. Chemokines such as CSF1 and CCL2, secreted by tumor cells, can recruit monocytes from peripheral blood to the TME, and then monocytes differentiate into macrophages. TAMs are a heterogeneous population which varies temporarily and spatially with the progression of the disease. They are the most plasticity and the highest proportion of immune cells in TME, and depending on the microenvironmental cues in the TME, they can undergo significant changes in their function. TAMs were generally divided into two polarization states: classically activated M1 and alternatively activated M2 subtypes. The M1/M2 macrophage paradigm plays a key role in tumor progression. M1 macrophages are historically regarded as anti-tumor, while M2-polarized macrophages contribute to many pro-tumorigenic outcomes in cancer through angiogenic and lymphangiogenic regulation, immune suppression, hypoxia induction, tumor cell proliferation, and metastasis. Abundant evidence suggests that TAMs are involved in anticancer signaling pathways, immunosuppressive, proangiogenic activities, and epithelial-to-mesenchymal transition (EMT) (Zhang et al., 2019a; DeNardo and Ruffell, 2019). It has recently been revealed that TAMs play an important role in the development of drug resistance in patients receiving anti-PD-1/PD-L1 therapy (Gordon et al., 2017). TAMs are plastic cells that adopt either anti-tumor (M1) or pro-tumor (M2) features to maintain homeostasis. M1-polarized TAMs are often detected in patients with early-stage cancers and are mediated by human interferons and endogenous/exogenous tumor necrosis factors (TNFs) to play a role in chronic inflammatory responses (Li et al., 2019a). In the TME, macrophages are always induced and transformed into the M2 phenotype, which is regarded as an important trigger of the immune escape mechanism as it supports the release of pro-tumor cytokines, promotes the formation of hypoxic TME, mediates EMT, and promotes cancer cell stemness and drug resistance, thereby contributing to tumor growth and progression (Vitale et al., 2019). As a new target, researchers are becoming increasingly interested in modulating TAMs for therapeutic purposes. Therefore it is crucial to clarify the relationship between TAMs and resistance to anti-PD-1/PD-L1 immunotherapy and elucidate the underlying molecular mechanism.

## 2 Effects of TAMS on HCC development

### 2.1 TAMs and tumorigenic factors

As a key player in anti-PD-1/PD-L1 immunotherapy, TAMs interact with tumorigenic factors and are involved in the development and progression of HCC. The majority of TAMs exhibit an M2 phenotype and is correlated with poor prognosis in a number of malignancies (Sica and Mantovani, 2012; Xu F. et al., 2020). Polarization of M2 macrophages is regulated by multiple cytokines in the TME. For example, overexpression of oxidored-nitro domain-containing protein 1 (NOR1) in TAMs is found to accelerate HCC progression by stimulating M2 polarization and TAMs-mediated inflammation (Chen et al., 2018). Colony-stimulating factor 1 (CSF1) secreted by HCC cells promotes overexpression of allograft inflammatory factor 1 (AIF1) in macrophages and induces a transformation of macrophages into the M2 phenotype, resulting in enhanced migration of tumor cells (Cai et al., 2017). TH2 cells secrete IL-4 and IL-13, cytokines that drive the polarization of the M2 phenotype (Cohen and Mosser, 2013). Malignant cells can secrete M2-like cytokines including IL-10, CCL2/3/4/5/7/8, CXCL12, VEGF and platelet-derived growth factor (PDGF), in an attempt to recruit a greater number of monocytes and M0 macrophages to the region and differentiate them into the M2 phenotype (Zhang et al., 2014). Hypoxia may be a key driver of macrophage recruitment and polarization in the TME. The high levels of chemokine, hypoxia inducible factor (HIF)-1/2, and endothelin-2 secreted by hypoxic tissues attract macrophages to areas of hypoxia (Murdoch et al., 2005; McGettrick and O’Neill, 2020). Damage associated molecular pattern (DAMP), specifically the high mobility group box 1 protein (HMGB1) is most commonly associated with hypoxia-induced macrophage polarization. It has been shown that HMGB1 is overexpressed in many solid tumors and correlates with the development of HCC (Liu Y. et al., 2015). Tumor cell-derived exosomes can also promote macrophage polarization toward M2 phenotype. For example, Wu et al. found that exosomes mediated transfer of functional CD11b/CD18 integrin (protein) from TAMs to tumor cells could potentially promote the migratory capability of HCC cells (Wu et al., 2021).

TAMs, which are a major component of the TME, can also promote tumor growth by secreting a variety of cytokines that induce immune suppression (Table 1). M2 TAMs play an important immunosuppressive role and have been found to secrete immunosuppressive molecules into the TME, including IL-10, transforming growth factor-β (TGF-β), Arg-1 and human leukocyte antigen G (HLA-G) (Komohara et al., 2016; Petty and Yang, 2017). Among them, TGF-β is noteworthy due to its strong immunosuppressive effect and close correlation with TAMs. TGF-β has been shown to affect anti-PD-1/PD-L1 immunotherapy by inhibiting the activation of T-cell and by inducing PD-L1 upregulation in tumor cells and tumor-associated angiogenesis. In HCC, TGF-β stimulates immune escape and tumor progression through TAMs recruitment (Mao J. et al., 2016). TGF-β has been demonstrated to promote the polarization of M1/M2 macrophages and plays a crucial role in neutrophil migration and cytotoxicity (Flavell et al., 2010; Santarpia et al., 2015). This suggests a potential therapeutic approach for HCC, which involves inhibiting the expression of TGF-β in TAMs. For instance, Sorafenib has been found to exhibit anti-tumor activities by reducing the level of TGF-β secreted by TAMs, thereby inhibiting liver cancer growth, metastasis, and EMT (epithelial-mesenchymal transition) (Fridlender et al., 2009; Wei et al., 2015). Furthermore, M2-type cells directly interact with myeloid-derived suppressor cells (MDSC) and actively suppress antitumor responses mediated by T cells (Parker et al., 2014). Tumor growth is driven directly by M2-like macrophages through the release of IL-1β, IL-6, IL-10, CCL18 and CCL20, as well as indirectly through the suppression of cytotoxic cell populations, including CD8⁺ T cells and NK cells (Arvanitakis et al., 2022). Guo et al. also provided evidence that infiltrating M2-TAMs were markedly elevated in the HCC TME, producing IL-17, a pro-inflammatory cytokine, and were augmented upon oxaliplatin treatment (Guo et al., 2017). TAMs containing a high level of CXCL8 can also promote cancer cell proliferation and metastasis (Yin et al., 2017). Recent publications have indicated that miRNAs derived from TAMs also confer drug resistance and immune escape in various cancers (Binenbaum et al., 2018; Sahraei et al., 2019; Ma et al., 2021). Therefore, a macrophage-targeted precision therapy should be considered, which may be the next step in reversing the drug resistance to PD-1/PD-L1 treatment.

**TABLE 1:** Factors secreted by M2-TAMs and their roles in tumor development.

Factors secreted by M2 TAMs	Pro-tumorigenic outcome
IL-6, EGF, TNF-α, IL-8, IL-10, CCL2, CCL18, CCL20, TGF-β, PDGF, STAT3, PI3K/AKT, BMP6	Tumor growth
IL-6, IL-10, IL-17, IL-1β, TGF-β, HLA-G, MMP-7, PD-1, PDE-2, CCL2, CCL5, Arg-1, TRAIL, Fas L	Immune suppression
CXCL-8, CCL3, CCL5, CCL18, CCL22, CCL20, MMPs, TGF-β, EGF, FGF, IGF-1, COX-2, TGF-β2, CSF-1, AIF1, COX-2, IL-10, IL-1β, Wnt	Tumor invasion and metastasis
VEGF-A, FGF, PDGF, PIGF, iNOS, COX-2, HIF, MMP-9, IL-10, Sp1, CXCR4, VCAM-1, FOXQ1, TIE-2	Tumor angiogenesis and lymphangiogenesis
TGF-β, MMPs, IL-6, IL-10, TNF-α, Cathepsin B, PI3K/AKT/mTOR, JAK-STAT3, miRNA	Anti-cancer therapy resistance

IL-6/8/10/17/1β, interleukin 6/8/10/17/1β; EGF, endothelial growth factor; TNF-α: tumor necrosis factor-α; CCL2/3/5/18/20/22: chemokine (C-C motif) ligand 2/3/5/18/20/22; TGF-β, transforming growth factor-β; PDGF, platelet-derived growth factor; JAK/STAT3, janus-activated kinase/signal transducer and activator of transcription 3; PI3K/AKT, phosphatidylinositde-3-kinase/protein kinase B; BMP6, bone morphogenetic protein 6; HLA-G, human leukocyte antigen G; MMP-7, matrix metallopeptidase 7; PD-1, programmed cell death protein 1; PDE-2, phosphodiesterase 2; Arg-1, arginase-1; TRAIL, TNF related apoptosis inducing ligand; Fas L, fas ligand; CXCL-8, chemokine (C-X-C motif) ligand 8; FGF, fibroblast growth factor; IGF-1, somatomedin C; COX2, cyclooxygenase 2; CSF-1, macrophage colony stimulating factor-1; AIF1, allograft inflammatory factor 1; VEGF-A, vascular endothelial growth factor-A; PlGF, placental growth factor; iNOS, inducible nitric oxide synthase; HIF, hypoxia-inducible factor; Sp1, specific β1 glycoprotein; CXCR4, chemokine (C-X-C motif) receptor 4; VCAM-1, vascular cell adhesion molecule 1; FOXQ1, forkhead box Q1; TIE-2, tyrosine kinase receptor with immunoglobulin and EGF homology domains-2; miRNA, microRNA.

### 2.2 TAMs and related signaling pathways

**FIGURE 1:**
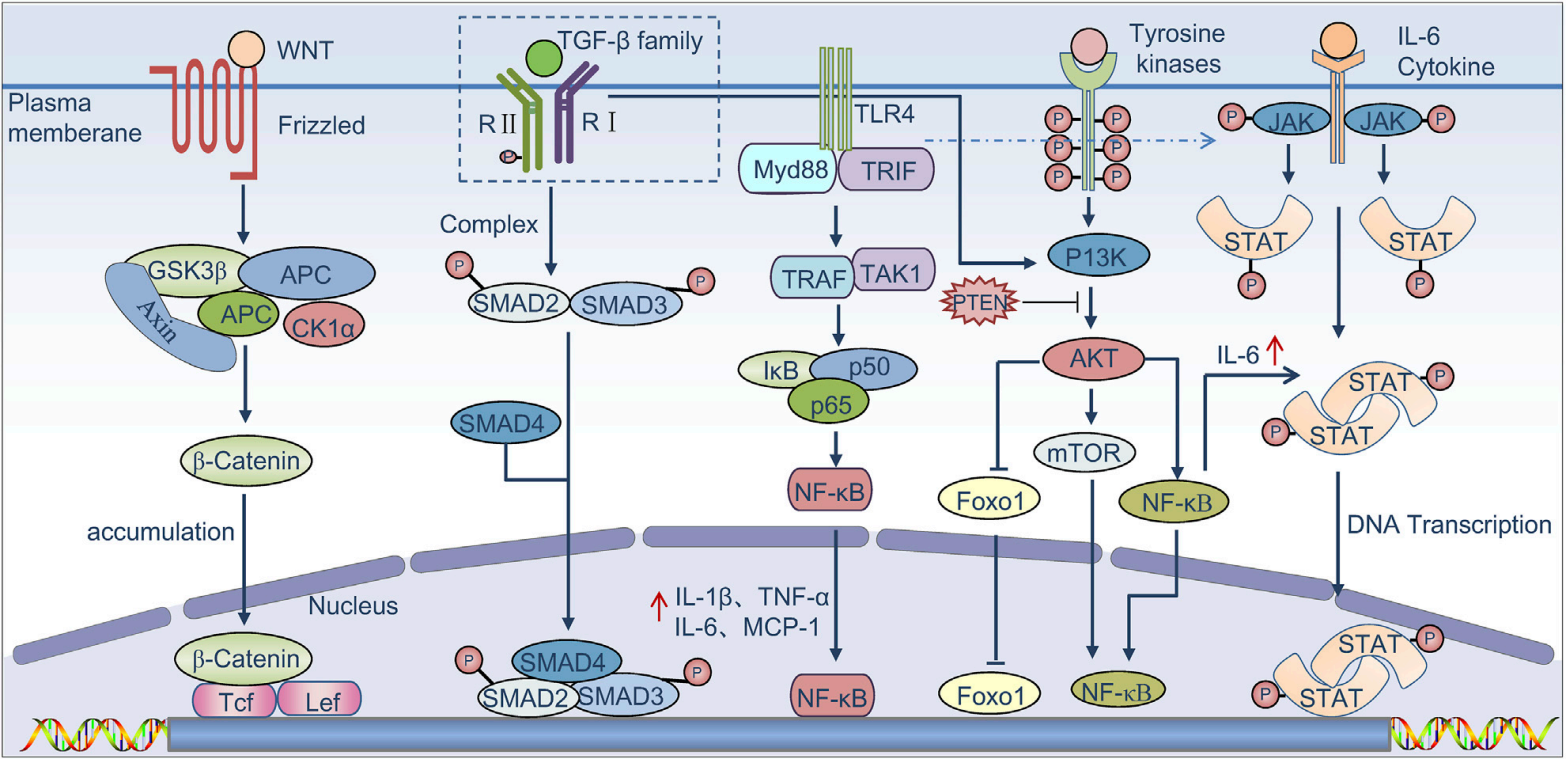
Signaling pathways that regulate TAMs to promote tumor progression. Binding of canonical WNT ligands to the Frizzled family of membrane receptors results in activation of the canonical WNT pathway. β-Catenin then translocates to the nucleus, resulting in the release of β-catenin from the GSK3β complex (glycogen synthase kinase-3β)–AXIN complex (axis inhibition protein)–APC complex (adenomatous polyposis coli protein), where it is known to bind to the transcription factors Tcf (T cell factor) and Lef (lymphoid enhancer-binding factor) in order to activate genes that promote the growth of tumor progenitor cells. TGF-β proteins bind to the TGF-β family of receptors, leading to phosphorylation of the receptors and activation of SMAD complexes, which induce immune suppression. The TGF-β pathway is also known to collaborate with the PI3K–AKT pathway, which in turn triggers activation of the mTOR complex and the nuclear factor-κB signaling axis (NF-κB). TLR4 binds to its ligand and can phosphorylate the IkB protein via TRIF, dissociate NF-κB from the trimer, promotes its nuclear transfer, and then promotes the release of factors such as IL-1β, TNF-α, and IL-6, which are known to promote tumor growth and induce immune suppression. In addition, the binding of several cytokines such as IL-6 to their receptors triggers the phosphorylation and activation of Janus kinases (JAKs) as well as the signal transducer and activator of transcription proteins (STATs); STAT dimers promote the proliferation and survival of tumor cells and induce immune suppression.

TAMs are involved in many signaling pathways contributing to HCC development, and the link between TAMs and tumor initiation has been extensively investigated in a variety of clinical samples and preclinical models (Figure 1) (Sica et al., 2008; Szebeni et al., 2017). For instance, active Wnt/β-catenin signaling in macrophages may promote the growth of tumor progenitor cells, thereby increasing the risk of HCC (Boulter et al., 2015; Debebe et al., 2017). Moreover, TGF-β/SMAD signaling pathway also plays an important role in immunosuppression, which can promote tumor cell escape through modulating the activity of immune cells, including macrophages and effector T cells (Batlle and Massagué, 2019). It is demonstrated that M2 macrophages promote the development and progression of HCC through the IL-6/STAT3 signaling pathway. Concurrently, the Toll-like receptor 4 (TLR4)/STAT3 pathway is also implicated, and this pathway can be activated by various hormones and growth factors via Janus kinase (JAK) activation. These activations lead to the phosphorylation and dimerization of STAT3, mediating its nuclear translocation and DNA binding, ultimately resulting in cell proliferation and survival (Wan et al., 2014; Yao et al., 2018; Li et al., 2019a). Activated STAT-3 in immunocytes features immunosuppressive activities, and STAT-3 at a high level is associated with a poor prognosis (Lu et al., 2018; Durham et al., 2019; Huang et al., 2019; Ren et al., 2019; Witt et al., 2021). TAMs-mediated upregulation of B7-H1 expression in HCC cells can be blocked by inhibiting the STAT-3 signaling pathway (Chen J. et al., 2012). It has been reported that overexpression of NOR1 in TAMs can accelerate diethyl nitrosamine (DEN)-induced HCC progression (Li et al., 2016; Chen et al., 2018). CCL17 expression in M2 macrophages can activate Wnt/β-catenin signaling and promote EMT of tumor cells, while tumor cells in turn activate TAMs through the TLR4/TRIF/NF-κB signaling pathway and increase IL-1β production mediated by HIF1α to trigger EMT (Zhu et al., 2016). Zhang et al. discovered that, under conditions of moderate hypoxia, M2 macrophages promote the secretion of IL-1β in HCC via the HIF-1α/IL-1β signaling loop, thereby enhancing tumor metastasis and contributing to the poor prognosis observed in HCC patients (Zhang J. et al., 2018). Recent studies have discovered that TAMs produce a large amount of ROS in response to massive upstream activation signals and mediate immunosuppression via the NF-κB pathway. It is inferred that the NF-κB pathway can modulate signal activation in cancer cells and tumor-infiltrating leukocytes to stimulate inflammation in the TME. Therefore, TNF-α blockage by anti-TNF-α antibodies is regarded as supportive of HCC treatment (Singh et al., 2019). The HIF1α signaling pathway plays a crucial role in the adaptation and progression of tumor cells in a hypoxic environment. It is demonstrated that in a hypoxic environment, the NF-κB pathway in macrophages is activated, leading to increased HIF1α expression (Cramer et al., 2003; Bandarra et al., 2015). Absence of HIF-1α can effectively inhibit macrophage motility and ECM migration (Krieg et al., 2000; Li et al., 2019b). Yue S et al. found that hepatocyte phosphatase and tensin homolog (PTEN) is quantitatively dominant and regulate liver immune response. Mice with a myeloid-specific PTEN knockout, which promotes liver PI3K/Akt/Foxo1 signaling, have been shown to exhibit increased levels of M2 macrophages and demonstrate lower TNF-α responses and higher IL-10 levels in response to TLR ligands, suggesting that PTEN plays a key role in macrophage differentiation (Yue et al., 2014).

### 2.3 TAMs and immunosuppressive TME

Immunosuppression is the key feature of TAMs biology. Infiltrating TAMs were found to be markedly elevated in HCC TME (Guo et al., 2017; Arvanitakis et al., 2022). The TAMs-induced TME mainly consists of immunocytes and mesenchymal stem cells (MSCs) that exhibit immunosuppressive properties and accelerate HCC progression (Li et al., 2019a). In the TME, M2-polarized TAMs are associated with tissue remodeling and inflammation inhibition, suggesting involvement of TAMs in HCC and their complex interaction with other immunocytes and MSCs. In HCC, high TAMs infiltration is positively correlated with T cells regulated by CD4, CD25, and FoxP3 and negatively correlated with CD8⁺ T cells. It is reported that blockade of colony stimulating factor 1 receptor (GSF1R) in TAMs can enhance CD8⁺ T cell infiltration while also reducing CD4⁺T cell infiltration (Ao et al., 2017). In addition to the effects on the densities of CD4⁺and CD8⁺ T cells, TAMs upregulate B7-H1 (PD-L1, CD274) expression in HCC cells, thereby suppressing the function of CD8⁺ T cells (Zhao et al., 2011; Chen J. et al., 2012). In the HCC TME, PD-L1 expression is mainly upregulated in Kupffer cells (KCs) instead of other antigen presenting cells (APCs) or tumor cells (Zhu et al., 2019). CD69⁺ T cells induce TAMs to produce indoleamine 2, 3-dioxygenase (IDO) and exert pro-tumor effects by releasing IFN-γ (Zhao et al., 2012). Additionally, decreases in T cell counts are associated with the FASL/FAS pathway (Muraoka et al., 2019; Cersosimo et al., 2020). In nonalcoholic steatohepatitis (NASH), IFN-γ is strongly correlated with inflammation, which imposes a risk of developing HCC; NKp46⁺NK cells can stimulate M1 polarization of TAMs. Therefore, NKp46⁺NK cells deficiency may twist the polarization of TAMs toward the M2-like phenotype and accelerate hepatic fibrosis progression (Tosello-Trampont et al., 2016). However, high infiltration of TAMs in HCC tissue is associated with reduced NK cell activity. TAMs are thought to induce early NK cell activation and subsequent dysfunction mediated by CD48/2B4 interactions (Wu et al., 2013). Sorafenib has been demonstrated to support the anti-tumor activities of NK cells by modulating the crosstalk between TAMs and NK cells (Sprinzl et al., 2013). T cells and NK cells are both crucial to tumor cell eradication, whereas other immune-related clusters (e.g., interaction between B cells and TAMs) play a modulating role in this process, which is infrequently addressed in related studies. Currently, there is merely one published study exploring the correlations of CXCR3⁺ B cells with IL-17-mediated inflammation and the polarization of pro-tumor macrophages in HCC (Liu R-X. et al., 2015).

### 2.4 TAMs promote angiogenesis and lymphangiogenesis in HCC

TAMs are a major driving force in tumor angiogenesis and lymphangiogenesis. Tumor angiogenesis is supported by TAMs primarily through the production of factors such as VEGFA, PDGF, PIGF, FGF, and TGF-β (Dhanasekaran et al., 2016; Llovet et al., 2016; Lai et al., 2019). Increased TAMs around TME are closely associated with poor prognosis in cancer patients, and M2 TAMs promote tumor growth and tumor metastasis by stimulating angiogenesis and lymphangiogenesis. Given the highly active metabolism in tumor cells and the metastatic properties of advanced tumors, angiogenesis and lymphangiogenesis are induced frequently for survival of the tumor cells. TAMs are essential to angiogenesis and lymphangiogenesis in HCC. Patients with HCC are shown to have a relatively high serum level of IL-23 secreted by inflammatory macrophages, which promotes macrophage-mediated angiogenesis by positively regulating the expression of IL-23 receptors on macrophages and accelerates HCC progression resulting from chronic hepatitis virus infection (Zang et al., 2018). Meng et al. discovered that macrophages accelerate the process of angiogenesis by modulating vascular CXCR4 expression via the ERK pathway (Meng et al., 2017). Peng et al. found that enrichment of TAMs in tumor cells is associated with microvascular density (MVD) in HCC (Peng et al., 2005). TAMs also secrete enzymes including CathepsinB, iNOS, COX2 and MMPs, which enhance angiogenesis via degradation of the extracellular matrix and invasion of endothelial cells (Ruffell and Coussens, 2015; Kumari and Choi, 2022). For example, TAMs can sense hypoxia in tumors, which can stimulate the chemotaxis of macrophages and increase the expression of MMP9 in TAMs, and finally promote the angiogenesis (Wu et al., 2021). Besides pro-tumor angiogenesis, TAMs are associated with lymphangiogenesis. Alishekevitz et al. found that TAMs promote lymphangiogenesis through the VEGF-C/VEGFR-3 pathway (Alishekevitz et al., 2016). Recently, Hwang et al. demonstrated that TAMs stimulate VEGF-A and VEGF-C in tumor cells, thereby promoting angiogenesis and lymphangiogenesis at tumor site (Hwang et al., 2020). It is demonstrated that TAMs promote lymphangiogenesis through transdifferentiation, direct penetration into the endothelial layer, and local stimulation of existing lymphatic endothelial cells (Iurca et al., 2020). The number of TAMs in the lymph nodes affected by tumor metastasis is shown to have a negative correlation with the prognosis of cancer patients (Oosterling et al., 2005). Macrophages can promote lymphangiogenesis through the secretion of growth factors and proteases that lead to the formation of lymphatic vessel (Gordon et al., 2010). Recent research found that IL-10 produced by hypoxic TAMs adjacent to lymphatic vessels was essential for the formation of lymphatic vessels by inducing the upregulation of Sp1 (specific β1 glycoprotein) in lymphatic endothelial cells (Chen et al., 2021). TIE-2 (Tyrosine kinase receptor with immunoglobulin and EGF homology domains-2) expressing TAMs are highly angiogenic and lymphangiogenic cells that exhibit immune-suppressive activity through secreting IL-10 and VEGF (Guex et al., 2015; Bron et al., 2016). Lymphangiogenesis leads to tumor metastasis, so the role of TAMs in regulation is important to understand and further investigate (Wang X. et al., 2021).

### 2.5 TAMs and EMT

EMT is an evolutionarily conserved developmental program. TAMs can promote EMT by secreting different cytokines and chemokines. This promotion mechanism has been extensively studied (Fan et al., 2014). The long-term interaction with macrophages results in poor migration and infiltration of CD8⁺ T cells within the tumor island, which makes PD-1/PD-L1 treatment resistant. TAMs secrete TGF-β in a manner similar to TGF-β secreted by cancer-associated fibroblasts (CAFs) and induce EMT (Fan et al., 2014). In addition, studies in xenograft nude mouse models of HCC indicate that M2 TAMs release TGF-β, mediate Smad2/3 binding to the miR-362-3p promoter, lead to miR-362-3p overexpression, and promotes EMT processes that significantly contribute to proliferation, invasion, and metastasis of HCC cells (Zhang et al., 2019b). EMT inducers such as TGF-β and receptor tyrosine kinase (RTK) ligands bring about changes in gene expression through an intricate signaling network, resulting in upregulated expression of C2H2 zinc finger (ZnF) proteins, zinc finger E-box binding homeobox 1 and 2 (ZEB1 and ZEB2/SIP1), and fundamental helix-loop-helix (bHLH) factors like TWIST and E47 (Peinado et al., 2007; Roy et al., 2021). These proteins bind to the E-box element situated in the promoter region of E-cadherin and recruit histone deacetylases (HDACs) and other factors to promote chromatin condensation and subsequent inhibition of E-cadherin transcription. Reduced E-cadherin expression can induce disruption of adherent junctions and other signaling events, such as functional regulation of Rho GTPases, eventually leading to loss of cell polarity (Cai et al., 2018). A normal EMT process is strictly dependent on gene expression, while tumor cells are prone to EMT for invasion and migration. TAMs are shown to play a role in the EMT process in tumor cells. CCL17 expression in M2 macrophages can induce EMT and activate the Wnt/β-catenin signaling pathway in tumor cells (Liu R-X. et al., 2015; Elia and Fallahi, 2017). Additionally, tumor cells activate TAMs through the TLR4/TRIF/NF-κB signaling pathway and upregulate HIF1α-mediated IL-1β production to stimulate EMT of tumor cells (Li et al., 2019a). Zhang et al. discovered that the HIF-1α/IL-1β feedback loop between tumor cells and TAMs contributes to EMT and *in vitro* metastasis of cancer cells in the hypoxic TME (Zhao et al., 2011). Recent studies have demonstrated that mesenchymal carcinoma cells induce TAMs formation via granulocyte-macrophage colony stimulating factor (GM-CSF) secretion. Once TAMs are formed, CCL18 is secreted, which induces EMT and promotes cancer metastasis (Su et al., 2014). TAMs can also secrete IL-6 which activates cyclooxygenase 2 (COX2), prostaglandin E2 (PGE2) and β-catenin signaling pathways, ultimately leading to EMT and cell invasion (Lu et al., 2014; Che et al., 2017). Thus, macrophage depletion could reactivate CD8⁺ T cells to migrate and invade tumor islands, and enhance the therapeutic effect of anti-PD-1 therapy.

## 3 The interaction between TAMs and the immune system in HCC

### 3.1 Interaction between TAMs and CD4⁺T lymphocytes promotes hepatocarcinogenesis

In addition to the major role in adaptive immune responses, CD4⁺ T cells are critical in modulating host immunity in response to different classes of pathogens. Upon activation, naïve CD4⁺ T cells differentiate into T helper (Th) effector cells, including Th1, Th2, Th17 and Tregs, and different subsets are shown to have distinct effects on cancer growth (Jia et al., 2015). Th1 cells are primarily characterized by their anti-tumor activity, whereas Th2 cells are associated with tumor-promoting effects. Treg cells suppress anti-tumor immune response by inhibiting the functions of multiple immune cells, including Th1 cells, CD8⁺ T cells, NK cells, and tumor-infiltrating DC cells. These cells affect the tumor TME by altering the direction of macrophage polarization. Th1 cells secrete IFN-γ to induce M1 polarization, and Th2 cells secrete IL-4, IL-5, and IL-10 to promote M2 macrophage production (Mills et al., 2017). TAMs, induced by tumor activated HIF1α, could secrete IL-23, thereby promoting the proliferation of Tregs and the expression of IL-10 and TGF-β. Additionally, they could inhibit cytotoxic lymphocytes from killing tumor cells (Fu et al., 2019). Additionally, TAMs overexpress CCL1, the ligand of CCR8 expressed on Tregs, to attract Tregs into the tumor area, inhibiting T cell immunity and promoting tumor growth. CD4⁺T cells at a reduced level are shown to promote HCC development (Brown et al., 2018). Moreover, Th17 and Treg cells are two subsets of CD4⁺T cells. Th17 cells are considered pathogenic effectors that contribute to disease exacerbation, while Treg cells suppress liver protection (Zhang H. et al., 2019). These findings indicate the critical role of CD4⁺ T cells in immune surveillance against HCC. Early studies have established a strong correlation between HCC development and chronic inflammation, highlighting the crucial role of TAMs in the pathogenesis of chronic liver disease due to their M1/M2 phenotype shifts. A study proposed that monocyte-derived TAMs exert an inhibitory influence on liver cancer progression by aiding CD4⁺ T cells in eliminating senescent liver cells (Kang et al., 2011). Conversely, another study revealed a shift in TAMs from anti-tumorigenic into pro-tumorigenic phenotype preceding the emergence of HCC. This shift triggers the production of chemokines (e.g., CCL17 and CCL22) and recruits of CD4⁺ Treg cells, ultimately leading to HCC development (Hefetz-Sela et al., 2014). Overall, in a chronic inflammatory environment, TAMs are susceptible to transformation into a pro-tumorigenic M2 phenotype, which promotes HCC development through the recruitment of CD4⁺ Treg cells.

TAMs promote HCC development and progress through recruitment of CD4⁺ T cells. In turn, CD4⁺ T cells accelerate tumor growth by releasing cytokines which in turn affect TAMs. For example, Katharina et al. discovered that CD4⁺CD25⁺Treg cells had direct inhibitory effects on the activation and functions of human monocytes/macrophages, which consequently impeded their functions as innate and adaptive immune effector cells (Giesbrecht et al., 2019). Wei et al. pointed out that activated CD4⁺ T cells in liver tissue stimulated CXCL10 production by macrophages to generate IgG-expressing plasma cells through the interaction of CXCL10 with CXCR3 on B cells (Wei et al., 2019). IgG is capable of activating the Fc receptor on macrophages and thereby inhibiting cytokine production during anti-tumor immune response. In the mouse model with HCC, the activation of macrophages was suppressed by the depletion of B cells, which enhanced the anti-tumor T cell response and inhibited HCC growth. In addition, DG Denardo et al. showed that CD4⁺ T cells amplified the pro-tumorigenic properties of macrophages by responding to IL-4 to modulate breast cancer metastasis to the lungs (DeNardo et al., 2009). In conclusion, TAMs promote tumorigenesis by interacting with CD4⁺ T cells, while CD4⁺T cells in turn act on TAMs to further enhance tumor immune escape.

### 3.2 Interaction between TAMs and CD8⁺ T lymphocytes promotes hepatocarcinogenesis

**FIGURE 2:**
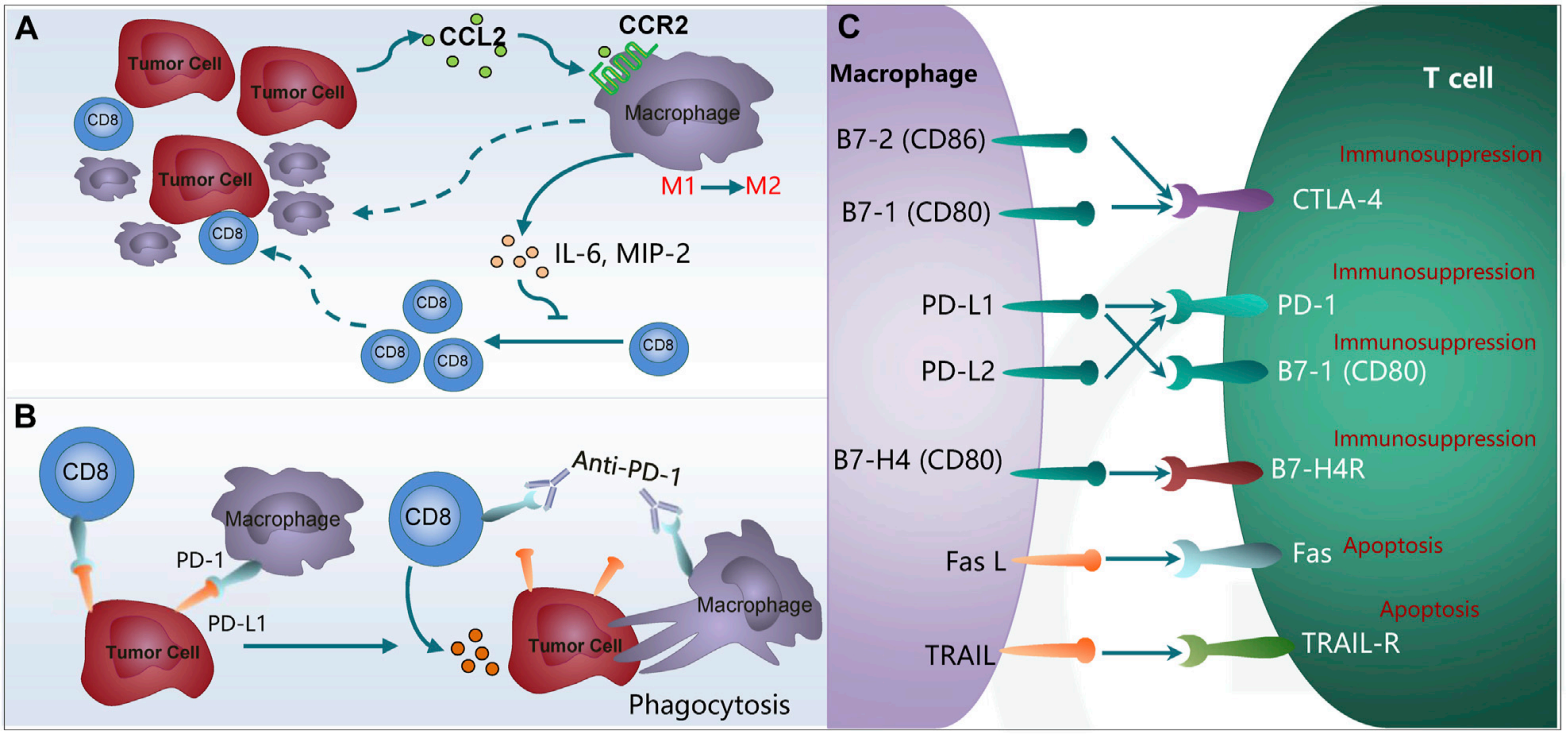
TAMs inhibit the immunotherapy through interaction with T cells. **(A)** High CCL2 expression correlates with more tumor-infiltrated TAMs and fewer CD8^+^ T lymphocytes. **(B)** Expression of PD-1 on the TAMs inhibits macrophages on phagocytosis and anti-tumor immunity. **(C)** Expression of PD-L1/L2, Fas L and TRAIL in M2 macrophages promotes T cell anergy and apoptosis. TAMs, tumor-associated macrophages; CCL2, chemokine (C-C motif) ligand 2; CCR2, chemokine (C-C motif) receptor 2; IL-6, Interleukin-6; MIP-2, macrophage inflammatory protein 2; PD-1, programmed cell death protein 1; PD-L1/L2, programmed cell death 1 ligand 1/ligand 2; CTLA4, cytotoxic T-lymphocyte-associated protein 4; Fas L, Fas ligand; TRAIL, TNF related apoptosis inducing ligand.

M2-like macrophages directly or indirectly promote tumor growth by inhibiting cytotoxic cells, including CD8⁺ T cells. CD8⁺ T cells are shown to produce opposite effects in a chronic pro-inflammatory microenvironment and exert antitumor surveillance. CD8⁺ cytotoxic T lymphocytes (CTLs) achieve target cell killing through direct cell-to-cell contact and interaction with the Fas receptor and its ligand FasL on the surface of cells as well as through the secretion of signals mediated by the synthesis of perforins and granzymes A and B (Ringelhan et al., 2018). However, there is a highly limited number of CD8⁺ T cells in the TME, which is correlated with activation of immune checkpoints such as PD-1 and CTLA-4. TAMs are shown to suppress the antitumor effects of CD8⁺ T cells by expressing ligands such as PD-L1 and CD80. Stress in the endoplasmic reticulum has been reported to trigger HCC cells to positively regulate PD-L1 expression in macrophages through exosome production and to inhibit T cell functions via the miR-23a-PTEN-AKT pathway (Liu J. et al., 2019). CC Chemokine Receptor 2 (CCR2) antagonists enhance the generation of CD8⁺ T cells by suppressing the immunosuppressive effect of TAMs in HCC (Yao et al., 2017). Besides immune checkpoints, TAMs release cytokines to inhibit CD8⁺ T cells proliferation and function. For instance, CCL2 released by HCC cells increases the number of M2 macrophages that inhibit CD8⁺ T cell proliferation by producing cytokines such as IL-6 and MIP-2 and thereby accelerate HCC progression (Li et al., 2017) (Figure 2A).

### 3.3 Interaction between TAMs and regulatory T cells promotes hepatocarcinogenesis

Regulatory T cells are a major immunosuppressive cell population and exerting various effects on tumor progression. A recent study utilizing the CIBERSORT algorithm assessed the relative abundances of 22 subsets of immune cells infiltrating tumors in 1,090 HCC instances, unveiling four distinct tumor clusters. Notably, tumors characterized by augmented regulatory T cell counts and depleted M1 macrophages were associated with a more dire prognosis (Yu et al., 2020). Interestingly, it was observed that M2 TAMs, rather than M1 macrophages, tended to foster the recruitment of regulatory T cells to the tumor microenvironment. Specifically, the infiltration of TREM-1^+^ TAMs within the hypoxic HCC microenvironment plays a pivotal role in conferring resistance to anti-PD-L1 therapy, accomplished through the recruitment of CCR6^+^ Foxp3^+^ regulatory T cells through CCL20 secretion (Wu et al., 2019a). These recruited or induced regulatory T cells can further potentiate the immunosuppressive capabilities of TAMs. Regulatory T cells facilitate the sterol regulatory element-binding protein 1 (SREBP1)-driven metabolic processes of M2 TAMs by suppressing the production of IFN-γ by CD8^+^ T cells (Liu C. et al., 2019). Consequently, regulatory T cells indirectly sustain the survival of M2 TAMs, thus establishing a positive feedback pool (Liu C. et al., 2019).

### 3.4 Interaction between TAMs and NK cells promotes hepatocarcinogenesis

In the TME of HCC, natural killer (NK) cells are less prevalent and functionally impaired (Park et al., 2020). A key regulatory mechanism that affects NK cell function is the intricate crosstalk that occurs between diverse immune cell types, including tumor-associated macrophages (TAMs), within the HCC TME (Sung and Jang, 2018). TAMs indirectly suppress NK cells through the release of cytokines, including interleukin-10 (IL-10) and transforming growth factor-β (TGF-β) (Sung and Jang, 2018). Furthermore, TAMs express CD48 in the HCC TME and may engage directly with the 2B4 receptor on NK cells, leading to dysfunction of these immune effectors (Wu et al., 2013). In contrast, M1 macrophages have the potential to enhance the overall count of activated intrahepatic NK cells in chronic liver diseases (Ma et al., 2017). This implies that by inducing the repolarization of TAMs to the M1 phenotype, they might stimulate NK cells to eliminate tumor cells.

## 4 The role of TAMs in resistance to anti-PD-1/PD-L1 immunotherapy in HCC

Effective therapy that blocks PD-1/PD-L1 interactions relies heavily on the function of T cells. Inhibitors targeting these checkpoints aim to disrupt the interaction between PD-1 and PD-L1, thereby preserving the anti-tumor properties of T cells, reversing immune evasion, and restoring their capacity to induce tumor cell death. However, any disruption in the immune functions of T cells can lead to the failure of PD-1/PD-L1 blockade therapy. Additionally, the activation of T cells is yet another pivotal process for the success of PD-1/PD-L1 blockade therapy. Despite blocking PD-1/PD-L1, tumor cells can still counter the immune checkpoints’ activity by activating additional inhibitory pathways. This occurs through the expression of other immune checkpoints and their ligands within the tumor’s immune microenvironment (Kwantwi, 2023). Given that TAMs are an important component in the TME, and there is intricate relationship between TAMs and the PD-1/PD-L1 axis, TAMs will play a pivotal role in the resistance to anti-PD-1/PD-L1. TAMs contribute to T cell dysfunction and exhaustion through the expression of various immunosuppressive factors, such as PD1 and PD-L1 expression, Fas L and TRAIL, FcγRI, and so forth (Morrissey et al., 2021; Pu et al., 2021).

### 4.1 TAMs contribute to PD-1/PD-L1 blockade resistance by inhibiting the migration of T cells activated by anti-PD-1 antibodies to the tumor islets

The primary purpose of anti-PD-1/PD-L1 immunotherapy is to restore T cell function (Chen and Mellman, 2017). However, there has been a lack of research focusing on promoting the migration of T cells to tumor nests. The extracellular stroma plays a crucial role in tumor growth and progression, with its main components including neovasculature, fibroblasts, and TAMs. Tumor extracellular stroma has been observed to inhibit the infiltration and activation of T cells (Cai et al., 2019). Numerous studies have confirmed that the exclusion of CD8^+^ T cells from tumor nests is consistently associated with a poor clinical response. TAMs have been shown to promote the formation of extracellular matrix (Afik et al., 2016), and the poor migration and infiltration of CD8^+^ T cells into tumor islets is a result of prolonged interaction with TAMs, which can trap lymphocytes (Peranzoni et al., 2018). TAMs control the recruitment of T cells and induce their exclusion within the tumor, thereby contributing to anti-PD-1/PD-L1 resistance (Kwantwi, 2023). Quaranta et al. have identified granulin as potentially involved in the specific mechanism of inhibiting the migration of CD8^+^ T cells to tumor nests mediated by TAMs (Quaranta et al., 2018). Moreover, TAMs can secrete granulin to exclude CD8^+^ T cells from metastatic livers, thereby promoting tumor progression. Genetic depletion of granulin has been found to reduce fibrotic stroma formation, thereby allowing T cells to migrate to metastatic sites and exert an anti-tumor effect (Nielsen et al., 2016). Consequently, the depletion of macrophages may reactivate the migration of CD8^+^ T cells and their invasion of tumor islets, ultimately improving the therapeutic effect of anti-PD-1 treatment.

### 4.2 TAMs contribute to PD-1/PD-L1 blockade resistance by inhibiting phagocytosis and anti-tumor immunity through expression of PD-1

PD-1 was first identified as an immune checkpoint receptor present on activated T cells, and its key role in suppressing T-cell activation upon binding with PD-L1 has been extensively established (Parry et al., 2005). However, it is noteworthy that PD-1 expression is not confined to T cells alone; it has also been reported to be expressed on other immune cells, including B cells (Sun et al., 2022) and NK cells (Hsu et al., 2018). Amongst these cells, TAMs constitute the predominant immune cells found within tumor islets. Recently, there has been a surge in research investigating the role of PD-1-expressing TAMs in tumor immune evasion. After interacting with its ligand PD-L1, it plays cardinal role in inducing immune evasion of cancer cells. Mouse and human M2 macrophages are both shown to express PD-1, with a later stage of cancer indicating a higher level of PD-1 expression and weaker phagocytic function of M2 macrophages (Gordon et al., 2017). PD-1/PD-L1 blockage *in vivo* can effectively enhance phagocytosis of macrophages, suppress tumor growth and achieve prolonged survival in tumor-bearing mouse models; moreover, a greater number of tumor-infiltrating macrophages in HCC tissue are indicative of longer survival. Gordon et al. found that the phagocytic potency of PD-1⁺ macrophages is rescued by the inhibition of PD-1/PD-L1 activity, which prolongs survival in preclinical colon cancer models in a macrophage dependent manner (Gordon et al., 2017; Li CW. et al., 2018). The researchers further suggested that the functions of PD-1^+^ TAMs are significantly compromised, and an increased proportion of PD-1^+^ macrophages aggravates the prognosis of patients (Li W. et al., 2022). This demonstrates that anti-PD-1/PD-L1 immunotherapy produces anti-tumor effects by acting on macrophages (Gordon et al., 2017). Besides, it is reported that the PD-1 expression level in M2 macrophages is negatively correlated with the anticancer activity, while PD-1/PD-L1 blockage *in vivo* is shown to enhance macrophage phagocytic capacity, suppressing tumor growth and prolonging the survival period of tumor bearing mice (Gordon et al., 2017; Hutchinson, 2017). M2 macrophages are suggested to inhibit T cell functions by releasing IL-17 to interact with PD-L1 in HCC (Chen J. et al., 2012). Ruan et al. also found that TAMs with high PD-1 expression tended to polarize toward M2-like macrophages, which may enhance the immune escape of tumor cells and may predict a poor prognosis (Ruan et al., 2021). The PD-1 overexpression significantly inhibited M1 macrophage polarization and phagocytosis as well as secreting more IL-10, which is a typical M2-related cytokine as an immunosuppressive cytokine. IL-10 has been shown to play an important role in promoting tumor immune evasion by decreasing the antitumor immune response in the tumor microenvironment (Mannino et al., 2015; Ham et al., 2017). These changes may enhance the pro-tumor effects of tumor microenvironment. In addition, anti-PD-1 therapy enhances the phagocytic ability of macrophages and the ability of macrophages to promote T cell activation by releasing immune activating factor, enhancing the anti-tumor immune response (Li CW. et al., 2018) (Figure 2B). On this basis, it is inferred that anti-PD-1 therapy may enhance the anti-tumor immune response of TAMs in HCC models by TAMs phagocytosis, which provides a new method for treating HCC of anti-PD-1 resistance.

### 4.3 TAMs contribute to PD-1/PD-L1 blockade resistance by promoting T cell anergy and apoptosis through the expression of PD-L1/L2

Besides PD-1, the expression of PD-L1 (B7-H1, CD274)/L2 (B7-DC, CD273) is detected in TAMs, which induce CD8⁺ T cells anergy and apoptosis through PD-1 binding on the surface of CD8⁺ T cells to inhibit anti-tumor immune response (Figure 2C). Furthermore, the expression of PD-L1was more frequently detected on immune cells, particularly TAMs, compared to malignant cells in HCC. Recent studies have also demonstrated that PD-L1 expression on TAMs is more closely related to clinical response to anti-PD-L1 treatment than PD-L1 expression on tumor cells (Kuang et al., 2009; Liu C. et al., 2019). In this condition, treatment with anti-PD-1/PD-L1 will be less effective. Kuang et al. were the first to show that macrophages are the predominant immune cells that express PD-L1 in HCC (Kuang et al., 2009; Wu et al., 2009). Recently study demonstrated that PD-L1 was preferentially expressed on CD68⁺ macrophages in the TME of HCC (Park et al., 2021). These PD-L1⁺TAMs, activated by tumor-derived IL-10, have been proposed to mediate CD8⁺ T cell dysfunction through the PD-1/PD-L1 interaction (Kuang et al., 2009; Wu et al., 2009). Reportedly, PD-1/PD-L1 protein-protein interaction mediates CD8⁺ T cell inhibition by resident hepatic macrophages (KCs) in HCC (Moreno-Cubero and Larrubia, 2016). Interestingly, PD-L1 overexpression in HCC is found to have correlations with TAMs (Chen J. et al., 2012). B7-1/CD80 and B7-2/CD86 in TAMs are shown to inhibit T cell activation by binding with T cell co-inhibitory receptor CTLA-4, which exhibits a higher affinity than the T cell co-stimulatory receptor CD28 (Wong et al., 2005). The above studies demonstrated that PD-L1 expression on TAMs plays a more critical role in suppressing CD8^+^ T cell effector function than tumor-derived PD-L1. PD-L1 and PD-L2 overexpression is observed in the TAMs of many cancer tissues. These ligands are shown to inhibit TCR (T cell receptor) signal transduction and promote T cell anergy and apoptosis by binding with PD-1 on CD4⁺ and CD8⁺ T cells (Lu et al., 2019). Additionally, B7-H4, another member of the B7 family, is also found in TAMs, which binds with the receptors on activated T cells to inhibit T cell proliferation, cytotoxicity, cell cycle progression and cytokine production (Kryczek et al., 2007). In addition to causing T-cell dysfunction, researchers also found that TAMs attenuated PD-L1 expression in tumors and inhibited T cell infiltration (Jeong et al., 2019). CD4 and CD8α T cell infiltration was found to be significantly increased after TAMs were depleted from tumors (Bloch et al., 2013). Numerous factors in the TME have also been shown to affect PD-L1 expression on TAMs, such as the IL-27/STAT3 axis induces PD-L1/2 overexpression in TAMs (Horlad et al., 2016). Progranulin could upregulate PD-L1 on TAMs through STAT3 pathway to promote T-cell exhaustion and immune evasion (Fang et al., 2021). TAMs also can induce PD-L1 expression by the secretion of IFN-γ through the JAK/STAT3 and PI3K/AKT/mTOR signaling pathways (Zhang et al., 2017). Furthermore, PD-L1-expressing TAMs are immunosuppressive and able to reduce the number of activated T cells through apoptosis (Prima et al., 2017). Taken together, these studies suggest that TAMs-derived PD-L1 in the TME of HCC is a major and important source for PD-L1/PD-1 axis–mediated suppression on intratumor CD8^+^ T cells, which further induce resistance to anti-PD-1/PD-L1 therapy. TAMs seem to be a novel target to resolve TAMs-related PD-1/PD-L1 blockade resistance (Li W. et al., 2022). In this case, if TAMs can be prevented from expressing PD-L1/L2, it will benefit the therapeutic effect of ICIs.

### 4.4 TAMs contribute to PD-1/PD-L1 blockade resistance by promoting T cell apoptosis through the expression of FasL and TRAIL

Both FasL (Fas ligand) and TRAIL (TNF-related apoptosis-inducing ligand) belong to the death ligand family. They are capable of binding to death receptors on the cell surface, such as the Fas receptor and TRAIL receptor, thereby initiating apoptotic signal transduction pathways. In M2 macrophages, when the expression of FasL and TRAIL increases, they can bind to the corresponding receptors on the surface of T cells. By inducing T cell apoptosis, they further suppress the activity of T cells, enhancing the immune escape capability of tumor cells and subsequently resulting in resistance to anti-PD-1/PD-L1 treatment. TRAIL demonstrates selective cytotoxic activities and induces apoptosis of tumor cells, transformants or virus infected cells without affecting normal cells (Huang et al., 2005). Therefore, TRAIL is regarded as a new path to anti-tumor therapies. However, recent studies have confirmed that M2 macrophages are shown to directly induce T cell apoptosis by expressing Fas L and TRAIL to demonstrate their immunosuppressive properties (Figure 2C). For example, Ritsuko et al. revealed that FasL expression and phagocytosis of M2 macrophages were modulated by enhanced NF-κB activation to accelerate T cell apoptosis, denoting the essential role of FasL expression in M2 macrophages to maintain T cell homeostasis in peripheral immune systems (Oura et al., 2013). Brown et al. demonstrated that induced TAMs can express FasL and release active soluble FasL in order to induce apoptosis of the Fas⁺lymphocyte (Brown and Savill, 1999), while CAFs (cancer associated fibroblasts) can upregulate FasL and PD-L2 via the cross-presentation of MHC-1 (major histocompatibility complex1) antigen and inhibit CD8⁺ T cells activity, thus elicit an immunosuppressive TME (De Jaeghere et al., 2019). Tsai et al. showed that TAMs expressed TRAIL to bind with corresponding receptors and mediate T cell apoptosis (Tsai et al., 2004). T cell activation is a critical process for PD-1/PD-L1 blockade therapy. Effective PD-1/PD-L1 blockade therapy relies on the T cell function, and any disruption in the processes of T cell immune function will result in the failure of PD-1/PD-L1 blockade therapy (Sun et al., 2020; Toor et al., 2020). In general, TAMs can directly promote T-cell apoptosis by expressing Fas L and TRAIL to further inhibit the activity of T cells, thereby enhancing the immune escape ability of tumor cells and leading to resistance to PD-1/PD-L1 therapy. Therefore, targeting Fas L and TRAIL expression in M2 TAMs may be a potential strategy to overcome anti-PD-1/PD-L1 resistance in cancer treatment. This is one of their important mechanisms for regulating immune response and affecting tumor development. This mechanism has important guiding significance for understanding the function of TAMs in the TME and developing tumor treatment methods targeting TAMs.

### 4.5 TAMs contribute to PD-1/PD-L1 blockade resistance by rapidly capturing anti-PD-1 from T cells and inducing an M2 phenotype through expressing FcγRI

**FIGURE 3:**
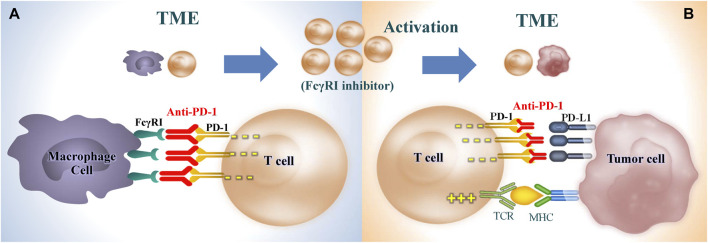
Restoration of T cell immunity by inhibiting FcγRI receptor on TAMs. **(A)** Inhibition of TAMs capturing anti-PD-1 from T cells in a FcγRI-dependent manner. **(B)** Restore the killing effect of T cells on tumor cells and enhance the efficacy of anti-PD-1. TME, tumor microenvironment; PD-1, programmed cell death protein 1; PD-L1, programmed cell death 1 ligand 1; FcγRI, CD64, Receptor I for the Fc Region of Immunoglobulin G; TCR, T cell receptor; MHC, major histocompatibility complex.

Unrelated to the previously mentioned mechanisms, it has recently been suggested that TAMs contribute to resistance against anti-PD-1 strategies through another mechanism that relies on Fc receptors and the elimination of monoclonal antibodies from T cells. Fcγ receptors (FcγRs) are a class of glycoproteins situated in the Fc domain of IgG and expressed on the membrane of immune cells, which link humoral and cell-mediated immunity by interaction with specific antibodies and effector cells. The Fc domain of PD-1 inhibitors is capable of binding with Type I FcR (FcγRI/CD64)-expressing cells, which appears to affect the efficacy of PD-1 inhibitors (Zhang T. et al., 2018). There are three reasons. First, the TME contains a high density of FcγRI⁺M2 macrophages, and aPD-1 antibodies bind with FcγRIs on the surface of M2 macrophages to induce antibody-dependent cellular cytotoxicity (ADCC), antibody-dependent cellular phagocytosis (ADCP), and complement-dependent cytotoxicity (CDC), resulting in T cell exhaustion, non-combat loss of T lymphocytes and eventually a significant reduction in-the anti-tumor activities induced by aPD-1 antibodies (Dahan et al., 2015). *In vitro* studies demonstrate that in FcγR-knockout mouse models, aPD-1 antibodies exhibit anti-tumor activities significantly higher than those without FcγR knockout, suggesting the inhibitory effects of FcγR on anti-tumor activities by binding with the Fc domain (Dahan et al., 2015; Chen et al., 2019). Small animal imaging results showed that in tumor-bearing mouse models injected with labeled aPD-1 antibodies, PD-1 effectively bound with tumor-infiltrating PD-1⁺CD8⁺T cells early after injection; however, TAMs subsequently scavenged the aPD-1 antibodies from T cells in a FcγRI-dependent manner to deactivate the antibodies (Arlauckas et al., 2017). Besides, T cells were preincubated with stained aPD-1 antibodies for 30 min and subsequently co-cultured with macrophages, following which a massive number of aPD-1 antibodies were detected in the macrophages; anti-PD-1 antibody transfer from T cells to macrophages was prevented by pre-addition of FcγR inhibitor (Arlauckas et al., 2017). Second, aPD-1 antibodies are identified to induce macrophage infiltration by binding with FcγR, which entails a risk of cancer hyperprogression. Existing studies show that accelerated tumor growth is observed in some patients who receive treatment with immune checkpoint inhibitors (Lo Russo et al., 2019). Further, a blockade of Fcγ receptors before PD-1 antibody treatment showed prolonged binding of anti-PD-1 antibodies and CD8^+^ T cells infiltration (Lo Russo et al., 2019). Third, FcγRI exhibiting high affinity for IgG4 is highly expressed in M2 macrophages. As a class of IgG4 antibodies, PD-1 inhibitors are shown to perpetuate the transformation of TAMs into the immunosuppressive M2 phenotype by interacting with FcγRI (Swisher et al., 2014). In addition, aPD-1 IgG4_S228P_ antibody stimulates macrophage-induced phagocytosis of PD-1⁺ T cells by mediating cross-linking between PD-1⁺ T cells and FcγRI⁺ macrophages, which indicates a shift from PD-1 blockage to the activation and induction of IL-10 gene expression, thereby suppressing T cell-mediated immune response (Zhang T. et al., 2018). In short, aPD-1 immunotherapies can achieve better efficacy with reduced side effects by inhibiting the expression of FcγRI on the surface of TAMs, which demonstrates significant clinical value and attracting prospects (Figure 3).

### 4.6 TAMs contribute to PD-1/PD-L1 blockade resistance by secreting IDO to inhibit T-cell functions

Indoleamine 2,3-dioxygenase (IDO) is renowned for its negative immunomodulatory properties, including the degradation of tryptophan and the generation of immune-modulating tryptophan metabolites. This enzymatic activity suppresses T-cell proliferation, ultimately leading to immunosuppression (Jürgens et al., 2009). Research conducted by Toulmonde et al. revealed that the majority of soft-tissue sarcomas (STS) and gastrointestinal stromal tumors (GIST) resistant to PD-1 blockade exhibited a substantial infiltration of M2 TAMs, which were found to express IDO (Toulmonde and Italiano, 2018). This suggests that the presence of these macrophages and their secretion of IDO may contribute significantly to the resistance observed in these tumor types. Furthermore, prior studies have illuminated the mechanisms underlying the immunosuppressive effects of IDO in the tumor microenvironment. Firstly, IDO expressed by APCs in tumor-draining lymph nodes can deactivate effective T cells, thereby reducing their antitumor activity (Cai et al., 2019). Secondly, the expression of IDO by tumor cells themselves suppresses the anti-tumor immune response, enabling immune evasion (Brandacher et al., 2006). Meanwhile, IDO has the ability to trigger macrophages to polarize towards the M2 phenotype. In essence, the secretion of IDO by macrophages can contribute to the creation of an immunosuppressive microenvironment, representing a pivotal mechanism in resistance to PD-1 blockade. Recently, a promising strategy for enhancing the effectiveness of tumor immunotherapy has emerged through the combined application of IDO inhibition and immune checkpoint blockade, as reported in studies (Zamarin and Postow, 2015; Tierney et al., 2020). Additionally, it has been observed that the secretion of IDO by M2 TAMs can lead to the polarization of M1 TAMs towards the immunosuppressive M2 subtype, thereby leading to resistance to PD-1/PD-L1 blockade (Wang et al., 2014). In a phase 2 clinical trial conducted by Toulmonde et al. (2018), a significant infiltration of M2 TAMs expressing IDO1 within tumor sites was demonstrated. Notably, the IDO1/kynurenine pathway plays a pivotal role in promoting the immunosuppressive phenotype of M2 TAMs, contributing to their resistance to PD-1 inhibition (Toulmonde et al., 2018). However, the mechanisms underlying PD-1/PD-L1 blockade resistance mediated by IDO derived from M2 TAMs remain incompletely understood in this study. Taken together, the secretion of IDO by M2 TAMs creates an immunosuppressive tumor microenvironment, serving as a crucial mechanism underlying resistance to PD-1/PD-L1 blockade. This understanding may pave the way for the development of novel therapeutic strategies that target IDO and its associated immunosuppressive pathways, ultimately enhancing the effectiveness of immunotherapy in cancer treatment (Toulmonde and Italiano, 2018).

### 4.7 The role of TAMs in anti-PD-1/PD-L1 immunotherapy for HCC caused by different etiology

HCC accounts for 90% of the incidence of liver cancer. Chronic hepatitis caused by hepatitis B virus (HBV) and hepatitis C virus (HCV) infection is a major risk factor for HCC. Much attention has been paid to the efficacy and safety of ICIs immunotherapy in HCC patients with chronic viral hepatitis. As a cornerstone, CheckMate-040 studies have shown that the objective remission rate (ORR) of Nivolumab (anti-PD-1) is 20% (El-Khoueiry et al., 2017). A systematic review study showed that the effective rate of ICIs treatment in the HCC cohort with HBV/HCV infection was 18.6%, which was similar to the CheckMate-040 study (Pu et al., 2020). In addition, efficacy survival indicators for the HBV/HCV infected cohort were not statistically significant compared to the non-HBV/HCV infected cohort in the subgroup of Asian patients with CheckMate-040 (Yau et al., 2019). Therefore, HCC patients may benefit from ICIs regardless of whether or not they are associated with HBV/HCV infection (Pan et al., 2022).

Other non-viral etiologies include chronic alcohol consumption and non-alcoholic fatty liver disease (NAFLD/MASH). Although the incidence of hepatitis virus-related HCC has been reduced in recent years by the HBV vaccine and HCV antiviral therapy, the incidence of HCC has been increasing, mainly due to strong links with alcohol abuse, obesity and diabetes. NAFLD has developed into one of the leading causes of HCC, and has become the fastest growing cause of HCC patients in the world (Anstee et al., 2019). The disease spectrum of NAFLD includes simple fatty liver, NASH (non-alcoholic hepatitis), liver fibrosis, cirrhosis and HCC. Compared with viral hepatitis-related HCC, NAFLD-HCC has a more insidious course, often with larger tumors and poorer prognosis (Leung et al., 2015). A recent study published in Nature involving 1,656 patients with advanced HCC showed that NASH-HCC patients did not benefit from ICIs treatment, possibly due to impaired immune surveillance of CD8⁺ T cells in NASH patients, resulting in poor response to ICIs and even induce MASH-HCC (Pfister et al., 2021). At the same time, the study found that patients with MASH-HCC who received ICIs had lower survival (11 cases, 8.8 months survival) than HCC patients with other causes (107 cases, 17.7 months survival) (*p* = 0.034). In response to the above problems, the researchers found that macrophage polarization is involved in the pathogenesis and progression of NASH. Macrophages in the liver (Kupffer cells) promote HCC initiation and progression under the long-term chronic inflammatory infiltration (Zhang P. et al., 2022). Using single cell transcriptome analysis, they identified two notable features of the hepatic immune microenvironment during the pathogenesis of NASH: macrophage expansion with molecular features of TAMs and induction of CD8⁺ T cell exhaustion. The induction of T-cell exhaustion may serve as a protective mechanism to limit further hepatic damage caused by the function of cytotoxic T cells (Dudek et al., 2021; Pfister et al., 2021). ICIs aggravate liver injury in diet-induced NASH mice, thus limiting immunotherapies targeting HCC. Unlike T-cells, TAMs undergo massive expansion during diet induced NASH. TAMs have been implicated in the context of liver tumorigenesis by orchestrating an immunosuppressive and tumor promoting microenvironment that facilitates the development of HCC. In summary, due to the impairment of T-cell function, PD-1 inhibition is less effective in patients with HCC complicated by NAFLD/NASH than in patients with viral hepatitis. The treatment based on TAMs is of great significance in the anti-PD-1/PDL1 treatment of NAFLD-HCC.

## 5 Strategies for overcoming resistance to anti-PD-1/PD-L1 blockade mediated by TAMs

Given that the complex biology of TAMs and their involved role in tumor proliferation, angiogenesis, EMT, metastasis, immune suppression, TAMs have a multichannel inhibitory effect on anti-PD-1/PD-L1 immunotherapy (Weiskopf and Weissman, 2015; Villanueva, 2017), so that a single ICI (Immune checkpoint inhibitor) cannot effectively activate the immune response. Therefore, targeting TAMs is of great significance to improve the efficacy of anti-PD-1/PD-L1 immunotherapy. There are several therapies available to recover the anticancer efficacy of PD-1/PD-L1 inhibitors by targeting TAMs, including eliminate TAMs already present in the TME, suppress macrophages recruitment, or reprogramming TAMs from the M1 to M2 phenotype (Table 2) (Kumari and Choi, 2022).

**TABLE 2:** Summary of studies evaluating the role of TAMs in HCC therapy.

Study (Year)	Product	Mechanism of action	Study subjects	Result
Li CW. et al. (2018)	nanoliposome-loaded C6-ceramide (LipC6)	Depletion of TAMs	CCl4 and TAg-transformed B6/WT-19 HCC cell line orthotopic mouse model	In HCC mice, administration of LipC6 reduced TAMs population and reduced their production of ROS as well as its ability to inhibit antitumor immune response
Xie et al. (2023)	Clodronate liposomes	Depletion of TAMs	PLC/PRF/5, MHCC97H, H22 and Hepa1-6 HCC cell line orthotopic mouse models	Suppressed tumor growth and metastasis
Zhang P. et al. (2022)	Doxorubicin-liposome and clodronate-liposome	Depletion of TAMs	diethylnitrosamine (DEN) induced primary HCC rat model	Depletion of macrophages by clodronate-liposome, in combination with Doxorubicin-liposome, significantly inhibited HCC progression compared with the use of Doxorubicin-liposome alone
Zhou et al. (2017)	Sorafenib and TACE	Termination of macrophages recruitment	Walker-256 HCC cell line xenograft and orthotopic rat models	Inhibition of tumor growth and angiogenesis
Teng et al. (2017)	CCR2 monoclonal antibody	Termination of macrophages recruitment	miR-122-knockout HCC mouse mode	Inhibition of tumor growth and activation of natural killer cells
Li et al. (2017)	CCR2 antagonist	Termination of macrophages recruitment	Hepa1-6 and LPC-H12 HCC cell line xenograft and Hepa1-6 orthotopic mouse models	Suppressed tumor growth and metastasis and reduced recurrence. Improved survival and activation of CD8⁺ T cells
Chen et al. (2022)	HMGA1 siRNA	Termination of macrophages recruitment	HepG2, Huh7, Hep3B, SNU-423, HCC-LM3, MHC C-97H, SK-Hep1, and SMMC-7721 HCC cell lines and SNU-423 orthotopic xenograft mouse models	Inhibition of HMGA1 expression reduced TAMs infiltration and Enhanced immunotherapy efficacy of CCL2-CCR2 signaling in HCC
Chen et al. (2014)	Sorafenib and CXCR4 antagonist (AMD3100)	Termination of macrophages recruitment	HCA-1 HCC cell line orthotopic mouse model	Blocking SDF-1α/CXCR4 reduced hypoxia-mediated HCC desmoplasia and increased the efficacy of sorafenib treatment
Chen et al. (2015)	CXCR4 antagonist	Termination of macrophages recruitment	HCA-1, JHH-7 or Hep3B HCC cell line orthotopic mouse models	Relieved regional immunosuppression and promoted anti-PD-1 treatment in a sorafenib-resistance HCC model
Ao et al. (2017)	CSF-1 receptor antagonist	Reprogramming polarization of TAMs	Hepa1-6, HepG2, or HCCLM3 HCC cell line orthotopic mouse models	Suppressed tumor growth and increased the activity of CD8⁺ T cells
Xu F. et al. (2020)	Listeria-based HCC vaccine (Lmdd-MPF)	Reprogramming polarization of TAMs	Hepa1–6/MPFG HCC cell line xenograft mouse model and clinical tissue samples collected from HCC patients	Restored the T-cell reactivity to the anti-PD-1 blockade
Wu et al. (2019a)	TREM-1 inhibitor (GF9)	Reprogramming polarization of TAMs	Hepa1–6 HCC cell line orthotopic mouse model and clinical tissue samples collected from HCC patients	Blocking TREM-1 with GF9 inhibitor reversed immunosuppression and anti-PD-L1 resistance in HCC
Yang et al. (2012)	17β-estradiol (E2)	Reprogramming polarization of TAMs	ANA-1 cells, Hepa1-6 HCC cell line and Heps HCC cell line xenograft and orthotopic mouse models	E2 inhibits alternative activation of macrophages and HCC progression by keeping ERβ away from interacting with ATP5J, thus suppressing the JAK1-STAT6 signaling pathway
Rodell et al. (2018)	TLR7/8-agonist-loaded nanoparticles	Reprograming polarization of TAMs	RAW 264.7 cell line, Murine bone marrow-derived macrophages (BMDMs) and xenograft mouse models	Promoted reprogramming of the TAMs and antitumor immunity
Wang X. et al. (2021)	CCL2 and CCL5 antibody (BisCCL2/5i)	Reprograming polarization of TAMs	clinical tissue samples collected from HCC patients, Hepa1–6 HCC cell line and orthotopic mouse models	The combination of BisCCL2/5i with anti-PD-L1 reduced immunosuppression in the TME and achieved long-term survival in mouse HCC models
Chang et al. (2020)	NanoMnSor to co-deliver sorafenib and MnO_2_	Reprograming polarization of TAMs	HCA-1, JHH-7 and Hep3B HCC cell lines and HCA-1 orthotopic mouse models	Reprogrammed tumor-promoting macrophages transition to immunostimulatory M1 macrophages, increased CD8⁺ cytotoxic T cells in tumors, and enhanced the efficacy of the PD-L1 antibody
Liu et al. (2020)	Selenium nanoparticles (SeNPs)	Reprograming polarization of TAMs	HepG2 HCC cell line	Promoting M2 to M1 macrophages and increasing the infiltration of natural killer cells into tumors

TAMs, tumor-associated macrophages; HCC, hepatocellular carcinoma; CCl4, carbon tetrachloride; ROS, reactive oxygen species; TACE, transcatheter arterial chemoembolization; CCR2, chemokine (C-C motif) receptor 2; HMGA1, high mobility group A1; CCL2, chemokine (C-C motif) ligand 2; CXCR4, chemokine (C-X-C motif) receptor 4; SDF-1α (CXCL12), chemokine (C-X-C motif) ligand 12; CSF-1, macrophage colony stimulating factor-1; TREM-1, triggering receptor expressed on myeloid cells-1; E2, estradiol; ERβ, estrogen receptor β; ATP5J, ATP synthase-coupling factor 6; JAK1, janus kinase 1; STAT6, signal transducer and activator of transcription 6; TLR7/8, toll-like receptor7/8; CCL5, chemokine (C-C motif) ligand 5; TME, tumor microenvironment; PD-1, programmed cell death protein 1; PD-L1, programmed cell death-ligand 1.

### 5.1 Eliminate TAMs already present in the TME

Given that the infiltration of TAMs restricts the clinically significant immune response of anti-PD1/PD-L1, eliminating them appears to be an appealing strategy. In early studies, clodronate-loaded liposomes (clodrolip) were often used to deplete liver macrophages (Van Rooijen et al., 1990). After injection, liposomes are artificially prepared vesicles that undergo phagocytosis by macrophages, and then intracellular delivery and accumulation of clodronate can induce macrophage apoptosis (Van Rooijen and Sanders, 1994). Li et al. demonstrated that nanoliposome C6-ceramide (LipC6) could enhance the anti-tumor immune response and hinder HCC growth in mice according to reducing the number of TAMs and their ability to suppress the anti-tumor immune response (Li G. et al., 2018). Xie et al. demonstrated that administration of clodronate liposomes partially depleted TAMs, resulting in reduced tumor growth and metastasis in a murine PLC/PRF/5, MHCC97H, H22 and Hepa1-6 cell- orthotopic tumor models (Xie et al., 2023). Similarly, Zhang et al. also found that compared with rats treated with Doxorubicin-liposome alone, macrophages depletion with clodronate-liposome (CL-LIP) in combination with Doxorubicin-liposome (Doxil) could significantly induce tumor cell apoptosis and thereby inhibit the progression of HCC (Zhang H. et al., 2022).

In recent years, researchers have found that Colony-stimulating factor-1 receptor (CSF-1R) blockers are the main way to deplete M2-TAMs (Gomez-Roca et al., 2019). When CSF-1 binds to its receptor, it initiates autophosphorylation, a vital process for macrophage proliferation, differentiation, and sustenance (Peranzoni et al., 2018). Therefore, employing a CSF-1R inhibitor (CSF-1Ri) may amplify the therapeutic potential of anti-PD-1/PD-L1 immunotherapy. PLX3397, a kinase inhibitor specifically tailored for CSF-1R, synergizes with anti-PD-1 therapy to enhance the infiltration and antitumor activities of CD8^+^ T cells within tumor tissues (Shi et al., 2019). Furthermore, it effectively alleviates tumor neovascularization and ascites formation (Dammeijer et al., 2017). Another notable CSF-1Ri, BLZ945, when combined with a PD-1/PD-L1 blocking antibody, also exhibits promising results in controlling tumor growth (Mao Y. et al., 2016). These existing antitumor drugs targeting CSF-1R present a novel avenue for integrated immunotherapy strategies. Some researchers have used CSF1R inhibitors to eliminate TAMs and redirect anti-PD-1 to CD8^+^ T cells, aiming to enhance the efficacy of combined therapy (Beider et al., 2014). A recent study explored the utilization of alginate-based hydrogel loaded with Pexidartinib (PLX)-encapsulated nanoparticles. These nanoparticles were designed to release PLX specifically at the tumor site, effectively interfering with the infiltration of TAMs. As reported by Li et al., regulating TAMs infiltration facilitated the infiltration of T cells, ultimately enhancing the local and systemic delivery of anti-PD-1 antibody-conjugated platelets. This approach aimed to prevent recurrence following surgical intervention (Li Z. et al., 2022). However, despite these preclinical successes, the persistence of the response to TAMs depletion is still lower than expected. In addition, compared to TAMs-targeted methods such as inhibiting TAMs recruitment or reprogramming, it has been found that TAMs depletion is less effective in preventing cancer growth.

### 5.2 Suppresses macrophages recruitment

Termination of macrophages recruitment is another treatment to combat the multi-faceted functions of TAMs in tumor progression and resistance. Zhou et al. demonstrated that ZA treatment enhanced the effects of hepatic transcatheter arterial chemoembolization (TACE) through inhibiting TAMs infiltration and tumor angiogenesis in rat models of HCC (Zhou et al., 2017). CCL2/CCR2 signaling plays a central regulatory role in circulatory monocytes and their infiltration into the TME. Teng et al. found that CCL2 is strongly upregulated in patients with HCC, and that gene silencing and administration of a neutralizing antibody to CCL2 or a CCR2 antagonist reduced recruitment of circulating monocytes, then decreased TAMs number, and downregulated M2 secretion function as TAMs. Finally, the liver damage, HCC incidence and tumor burden were significantly reduced (Teng et al., 2017; Yao et al., 2017). Li et al. found that blockade of the CCL2/CCR2 axis inhibits the recruitment of M2-TAMs, leading to a reversal in the status of immunosuppression in HCC (Li et al., 2017). A CCR2 antagonist, naturally occurring in Abies georgei and designated as 747, exhibited antitumor activity in mouse models of HCC. This was evident through a reduction in TAM levels and a concurrent increase in CD8 T-cells within the tumor microenvironment (Yao et al., 2017). Furthermore, studies revealed that concurrent administration of anti-CCL2 antibodies improved the effectiveness of chemotherapy and checkpoint inhibitors (Brana et al., 2015). Additionally, combining the CCR2 antagonist, RS504393, with anti-PD-1 therapy resulted in a superior tumor response compared to anti-PD-1 monotherapy in various murine tumor models. This combination therapy achieved an enhanced antitumor response by simultaneously boosting CD8^+^ T-cell recruitment and activation while reducing CD4^+^ regulatory T-cells. Chen et al. demonstrated that high-mobility group A1 (HMGA1) as a crucial regulator of macrophage recruitment through the activation of NF-κB-CCL2 signaling in HCC (Chen et al., 2022). Another key pathway in macrophages recruitment is the CXCL12/CXCR4 interaction. The upregulation of stromal cell derived factor 1alpha (SDF-1α/CXCL12) induced by hypoxia also contributes to the recruitment of suppressive M2 macrophages (Chen et al., 2014). Using the CXCR4 antagonist AMD3100, Chen et al. found that inhibition of the SDF-1α receptor (CXCR4) relieved regional immunosuppression and facilitated treatment with anti-PD-1 antibodies in a model of HCC resistant to sorafenib (Chen et al., 2015).

### 5.3 Reprogram TAMs from M1 to M2 phenotype

Recent findings suggest that re-education of M2 TAMs, rather than their depletion, could be a more efficient strategy. Previous research has indicated that the protumor M2 phenotype can be successfully re-educated to the tumoricidal M1 phenotype, effectively inhibiting the supportive role of TAMs in tumors. This approach may enhance the response to anti-PD-1 treatment (Kwantwi, 2023). As CSF-1R has been observed to be expressed in a restrictive manner by TAMs and monocytes that prefer to locate at the boundary of HCC (Jiang et al., 2020). CSF-1 and its receptor, CSF-1R, have been shown to regulate macrophage differentiation and function and to play an important role in macrophage infiltration in HCC (Ao et al., 2017). Targeting CSF1/CSF1R signaling in protumoral TAMs represents an attractive strategy for eliminating CSF1R-dependent or reprogramming M2-like TAMs in HCC (Pyonteck et al., 2013; Cannarile et al., 2017). Ao et al. found that treatment with the CSF-1R inhibitor PLX3397 resulted in delayed tumor growth in both xenograft and allograft models in HCC, and this delay was probably mediated by the transition from M2 to M1 macrophages in a population of TAMs induced by a block in the CSF1/CSF-1R signaling pathway (Ao et al., 2017). Xu et al. demonstrated that the Lmdd-MPFG (LM) vaccine activates the NF-κB pathway in TAMs through the TLR2-MyD88 pathways (Chen Y. et al., 2012; Xu G. et al., 2020), which skewed the TAMs from M2 into M1, and skewed the TME cytokine profile of the TME toward an anti-tumor profile, this change restored the T cell reactivity to anti-PD-1 blockade (Xu G. et al., 2020). Wu et al. demonstrated that HIF-1α induced increased expression of the triggering receptor expressed on myeloid cells-1 (TREM-1) in TAMs, leading to immunosuppression. Blocking TREM-1 treatment with the GF9 inhibitor reversed immunosuppression and resistance to anti-PD-L1 in HCC (Wu et al., 2019b). In addition, a study by Yang et al. identified that 17β-estradiol (E2) could attenuate the progression of HCC through the regulation of macrophage polarization, as E2 rechallenge reduced tumor growth in both orthotopic and ectopic mouse models of HCC, which functions as an inhibitor of alternative macrophage activation and tumor progression by keeping the estrogen receptor-β away from interacting with ATP5J and by interfering with the JAK1-STAT6 signaling pathway (Yang et al., 2012). Ubiquitin-specific protease 7 (USP7), an enzyme responsible for deubiquitination, holds significant promise as a therapeutic target due to its regulatory function in DNA damage repair and epigenetic inheritance (Wang et al., 2019). USP7 has been recognized as a gene that is highly expressed in M2 TAMs. Notably, specific inhibition of USP7 has the potential to reprogram M2 TAMs into M1 phenotype via the P38 MAPK pathway (Dai et al., 2020). Furthermore, targeting USP7 has been demonstrated to enhance the infiltration and cytotoxic activity of CD8^+^ T cells within the TME, while concurrently suppressing the expression of PD-L1 in tumor cells, thereby enhancing the response to anti-PD-1/PD-L1 therapy (Dai et al., 2020; Wang Z. et al., 2021).

Emerging studies have used nanoparticles to enhance drug delivery into TAMs to facilitate polarization to M1 phenotype. For example, Systemic targeting of TAMs with nanomedicines is an attractive approach because TAMs are ideal therapeutic targets due to their considerable propensity for phagocytose of nanoparticles (Han et al., 2021; Kumari and Choi, 2022). Recent studies have used nanoparticles containing TLR agonists or tumor peptides to promote TAMs reprogramming, utilizing nanoparticles to simultaneously target TAMs and promote anti-tumor immunity. For example, Rodell et al. found that nanoparticles loaded with the TLR7/TLR8 agonist R848 preferentially accumulated in TAMs in mouse models and promoted the polarization of cells towards M1 macrophage re-education (Rodell et al., 2018). Clinical trials are also underway for TLR agonists to treat solid tumors. TLR9 agonists, such as lefitolimod, effectively modulate the TME and induce anti-tumor responses by promoting the infiltration of CD8 T-cells and reprogramming TAMs in the TME (Kapp et al., 2019). In addition, the nanoparticle-based delivery of TLR agonists, bisphosphonates, DNA, mRNA, and miRNA repolarizes TAMs more effectively. Wang et al. encapsulated mRNA which can bispecifically bind and neutralize CCL2 and CCL5 (BisCCL2/5i) in a clinically approved lipid nanoparticle platform. The nanoplatform of This BisCCL2/5i mRNA significantly induced TAMs polarization from M2 to M1 and reduced the immunosuppression of the TME. Morever, BisCCL2/5i combination with anti-PD-L1 resulted in long term survival in mouse HCC models (Wang Y. et al., 2021). Chang et al. designed a NanoMnto tumor-targeted and biodegradable nano-delivery system to deliver sorafenib and MnO_2_. NanoMnSor reprograms the immunosuppressive TME by reducing the hypoxia-induced tumor TAMs infiltration, promote macrophage polarization to the M1 immunostimulatory phenotype, and increase the number of CD8⁺ cytotoxic T-cell numbers in tumors, thereby increasing the efficacy of the anti-PD-1 antibody, synergistically inhibited the progression of both primary HCC as well as distant metastases (Chang et al., 2020). Another recent study, selenium nanoparticles (SeNPs) were combined with cytokine induced killer cells (CIK), which effectively enhanced natural killer cell infiltration into tumors and reprogrammed tumor promoting M2 macrophages into anti-tumor M1 macrophages, stimulating a robust anti-tumor response to combat progression (Liu et al., 2020). Targeting TAMs offers a new approach to improving immunotherapy for HCC by harnessing the immune system. However, to obtain accurate therapeutic targets, further understanding of the molecular mechanisms controlling phenotypic changes in different processes of liver cancer is still needed.

## 6 Conclusion

HCC is a major public health threat that responds well to immunotherapy as an emerging treatment option. However, the evolution of drug resistance and the presence of immunosuppressive cells in the TME impose a challenge to sustain the efficacy of anti-PD-1/PD-L1 immunotherapy for HCC. M2 macrophages play a major role in achieving immunosuppression in the TME of HCC. Through interaction with different tumor-associated factors and cells, M2 macrophages can promote tumor angiogenesis and lymphangiogenesis, EMT, and drug resistance, revealing the mechanism responsible for the negative regulation of immunotherapy for HCC. TAMs affect anti-PD-1/PD-L1 immunotherapy for HCC in many ways. To be specific, TAMs interact with T cells, suppress phagocytosis and anti-tumor immunity through PD-1 expression, amplify T cell anergy and apoptosis through PD-L1/L2 expression, mediate T cell apoptosis through FasL and TRAIL expression, scavenge aPD-1 antibodies from T cells and transform into M2 phenotype polarization through FcγRI expression. Therefore, personalized immunotherapy for HCC can be developed by reference to the mechanisms responsible for the immunosuppressive activities of TAMs and related factors, such as blockage of PD-1, PD-L1/L2, Fas L and TRAIL, and FcγRI expression in TAMs, in order to enhance the efficacy of anti-PD-1/PD-L1 immunotherapy and improve the survival rate. Increasing evidence suggest that potential mechanisms of TAMs mediated tumor immune escape imply interpretation and breakthrough to bottleneck of current tumor immunotherapy (Qiu et al., 2021). TAMs appear to be an important potential target for cancer therapy, also new possibilities for improving the efficacy of anti-PD-1 monoclonal antibodies (Bouhlel et al., 2007; Orecchioni et al., 2019; Wang C. et al., 2021). Despite all that, the immunosuppressive effects of TAMs in the TME and the related mechanisms are not yet fully understood, and thus further explorations and discussions are needed to confirm the synergistic effect of TAMs targeted drugs and anti-PD-1 monoclonal antibody, and develop potential new strategies to overcome TAMs related immune tolerance. Furthermore, the differences among TAMs function in different tumors are primarily attributed to variations in their origins, proportions, and anatomic locales. Therefore, future investigations related to the development of TAMs-targeted therapies should be narrowed down to a specific tumor type, such as HCC. In the future, the customization of TAMs-targeted treatments in combination with anti-PD-1/PDL1 therapeutic approaches would be a promising alternative treatment strategy for HCC patients.
